# Enhanced pitch centering in individuals with laryngeal dystonia

**DOI:** 10.3389/fnhum.2026.1688947

**Published:** 2026-02-26

**Authors:** Rabab Rangwala, Anantajit Subrahmanya, Kurtis Brent, Saloni Gupta, Kamalini Ranasinghe, Corby L. Dale, Jessica L. Gaines, Alvincé L. Pongos, Clark A. Rosen, Sarah L. Schneider, Julie Barkmeier-Kraemer, Kristina Simonyan, John F. Houde, Srikantan S. Nagarajan

**Affiliations:** 1 Department of Radiology and Biomedical Imaging, University of California, San Francisco, San Francisco, CA, United States; 2 Department of Electrical and Computer Engineering, University of California, Santa Barbara, Santa Barbara, CA, United States; 3 University of California, Berkeley – University of California, San Francisco Graduate Program in Bioengineering, San Francisco, CA, United States; 4 Department of Neurology, University of California, San Francisco, San Francisco, CA, United States; 5 Department of Otolaryngology - Head and Neck Surgery, University of California, San Francisco, San Francisco, CA, United States; 6 Department of Otolaryngology - Head and Neck Surgery, The University of Utah, Salt Lake City, UT, United States; 7 Department of Otolaryngology - Head and Neck Surgery, Massachusetts Eye and Ear, Harvard Medical School, Boston, MA, United States

**Keywords:** auditory feedback, laryngeal dystonia, pitch centering, speech motor control, speech perception, speech production

## Abstract

**Introduction:**

Laryngeal dystonia (LD) is a neurological voice disorder marked by strained voice quality, pitch instability, and sudden voice breaks, yet the mechanisms underlying impaired vocal control are poorly understood. One key process, known as pitch centering, reflects the central nervous system’s ability to correct early pitch deviations during an utterance by converging toward an intended target. While pitch centering provides a sensitive window into the neural control of spontaneous speech, it remains unexamined in patients with LD and is presumed to contribute to disordered regulation of voice production.

**Methods:**

Here, we examined pitch centering in 24 individuals with LD [adductor LD (*n* = 20), abductor LD (*n* = 3), or both (*n* = 1)] compared to 29 healthy controls. The primary outcome measures were: (1) *Pitch centering* and (2) *Pitch movement*. *Pitch centering* was defined as the difference in the absolute values of initial (0–50 ms) and mid-trial (150–200 ms) pitch. Positive values (centering > 0) indicated a shift toward the median pitch defined as centering trials. *Pitch movement* was defined as the difference between mid-trial and initial pitch. In a subset of trials, we observed negative values of centering reflecting movement away from intended pitch targets, which we defined as *anticentering* trials. An additional subset of trials was defined as *overshoot trials*, instances where the normalized pitch movement crosses the median pitch at mid-trial.

**Results:**

Initial pitch deviation (*p* < 0.0001) and pitch movement magnitude (*p* < 0.0001) were significantly greater in individuals with LD compared to controls across all trials. Importantly, individuals with LD exhibited more pronounced centering responses compared to controls, with greater centering magnitude observed by a significant group-by-tercile interaction (*p* = 0.028). Individuals with LD and controls showed similar distributions of centering and anticentering trial types. However, LD patients exhibited significantly greater centering magnitude compared to controls across each trial type.

**Discussion:**

These findings offer valuable insights into speech motor and predictive control processes in LD, with potential implications for clinical assessment and treatment strategies aimed at improving patient quality of life.

## 1 Introduction

Laryngeal dystonia, previously termed spasmodic dysphonia, is a rare neurological movement disorder characterized by involuntary spasms of the laryngeal muscles resulting in intermittent vocal fold disruptions that impair voice quality (Lee et al., 2024; Mor et al., 2018). While its exact etiology remains unknown, recent studies suggest that a combination of genetic susceptibility, environmental influences, and maladaptive neuroplasticity may contribute to its complex pathophysiology. Most individuals with LD present with the adductor type, marked by excessive vocal fold closure, or hyperadduction. These physiological disturbances manifest clinically as pitch instability, sudden voice breaks, and a strained, effortful vocal quality during speech. Importantly, LD is a task-specific disorder, selectively affecting voluntary speech production while sparing other vocal behaviors such as whispering, laughing, crying, and yawning (Simonyan et al., 2021).

Neuroimaging studies in LD have identified central nervous system abnormalities that may contribute to behavioral voice symptoms, including reduced activity in regions involved in movement selection and motor preparation (Kothare et al., 2022). These findings further imply deficits in sending motor commands to the larynx from M1 and in generating predictions about their somatosensory consequences. Cerebellar activation abnormalities suggest impairments in feedback prediction. Additionally, post-voice onset hyperactivity in the somatosensory cortex and inferior parietal lobule, commonly reported in LD, suggests impairments in auditory and somatosensory feedback processing. These neural disruptions in motor planning, state prediction, and sensory feedback may contribute to broader alterations in functional connectivity across motor, sensory, and integrative networks. As a result, individuals with LD may overcompensate or under correct in response to sensory input, producing unstable or exaggerated vocal responses. Such symptoms may stem from inaccurate internal estimates of the laryngeal system’s state, faulty predictions about the sensory consequences of vocal motor commands, and a reduced ability to generate appropriate corrective responses (Houde and Nagarajan, 2011), ultimately leading to the unstable pitch, voice breaks, and vocal strain that characterize the disorder.

Understanding these deficits in neurological disorders such as LD requires situating them within the broader theoretical framework of speech motor control, as speech production is a complex process that depends on tightly coordinated neural control. Certain models of speech motor control describe the system as operating primarily through distinct feedforward and feedback mechanisms—where the central nervous system (CNS) issues motor commands to the lower motor system, which subsequently executes tasks and manages feedback responses. In this framework, feedback control is considered peripheral and largely reflexive. More recent theories, however, suggest a more dynamic and interactive role for the CNS in both feedback and feedforward speech motor control. The DIVA (*Directions Into Velocities of Articulators*) model emphasizes a framework in which mappings between motor commands and their expected sensory outcomes are a developmental process. It incorporates distinct neural maps within the CNS that guide motor execution and support error-based learning to refine speech targets over time (Tourville and Guenther, 2011). Conversely, the state feedback model of speech production posits that the CNS continuously estimates the evolving state of a movement and adjusts motor output based on both predicted and actual sensory feedback (Houde and Nagarajan, 2011). Prediction errors arising from mismatches between expected and actual feedback drive real-time motor corrections, highlighting the CNS’s central role in sensorimotor integration.

To further investigate how the speech motor system responds to perceived errors, researchers have employed altered auditory feedback paradigms in both healthy individuals and clinical populations, including those with Alzheimer’s disease (Ranasinghe et al., 2017), Parkinson’s disease (Liu et al., 2012), cerebellar ataxia (Gaines et al., 2024; Houde et al., 2019), and laryngeal dystonia (Kothare et al., 2022; Thomas et al., 2021). One commonly used method is the pitch perturbation paradigm, in which participants are asked to sustain a vowel sound while their auditory feedback is covertly shifted in pitch, either upward or downward, at an unpredictable time for a brief period. Studies show that speakers typically respond by adjusting their pitch in the direction opposite to the perturbation, indicating an active feedback control mechanism geared toward maintaining vocal stability (Houde and Nagarajan, 2011). While pitch responses to externally altered auditory feedback have been well studied, the mechanisms underlying inherent, self-generated pitch corrections during natural speech production remain underexplored.

Recent studies suggest that the CNS can also detect and correct subtle deviations in pitch during the early stages of spontaneous speech production. This process, referred to as *pitch centering*, occurs early on in an utterance and reflects the system’s ability to stabilize vocal output over time (Subrahmanya et al., 2024; Niziolek et al., 2013). Centering is quantified by tracking the pitch of a number of repetitions of a vowel utterance, computing the median pitch track, and then, for each utterance, comparing the value of pitch relative to the median at phonation onset with that at a later point within the same utterance. Speakers subconsciously respond to initial deviations from the median with pitch adjustments that converge toward the median pitch track as production of the utterance progresses. This convergence to the median suggests that each production of the vowel has an intended target pitch, and also demonstrates the CNS’s capacity for fine-grained, online motor correction. Notably, centering may occur independently of auditory feedback, suggesting a broader sensorimotor basis (Parrell et al., 2021). While this phenomenon has been shown to be impaired in neurological disorders such as Alzheimer’s disease (Subrahmanya et al., 2024), its role in clinical populations with voice-specific motor disruptions, such as laryngeal dystonia (LD), remains largely unexamined.

As pitch centering relies on precise state estimation and the ability to implement rapid pitch corrective responses (Niziolek et al., 2013), it serves as a sensitive behavioral window into the integrity of impaired sensorimotor mechanisms (Subrahmanya et al., 2024; Niziolek et al., 2013). Laryngeal dystonia, with its characteristic neural impairments, motor speech disruptions, and feedforward and feedback control deficits, provides an ideal model for examining these processes. Investigating pitch centering in LD not only offers valuable insight into speech motor control and predictive control processes in individuals with neurological voice disorders but also holds potential for identifying behavioral markers that can inform diagnosis and guide targeted interventions. In this novel study, we examined pitch centering in individuals with LD compared to healthy controls, to better understand the underlying mechanisms of impaired vocal control in this population. Given the known impairments in LD, we hypothesized that individuals with LD would demonstrate altered pitch centering, potentially reflecting maladaptive or exaggerated compensatory strategies relative to controls.

## 2 Methods

### 2.1 Participants

We included 24 patients with LD (16 Female, mean age = 54.1 years, standard deviation = 10.5) and 29 healthy controls (16 Female, mean age = 55.6 years, standard deviation = 14.6) from historical datasets of previous and ongoing research projects. The LD datasets were derived from studies on neural imaging during phonatory events in individuals with LD (Kothare et al., 2022) and an ongoing clinical trial on LD pathophysiology. Control subjects were recruited from these studies and a behavioral study on Alzheimer’s disease (Subrahmanya et al., 2024; Ranasinghe et al., 2019). All participants were recruited from the University of California, San Francisco (UCSF) Voice and Swallowing Center, the Memory and Aging Center, and the National Spasmodic Dysphonia Association’s website postings. A two-sample heteroscedastic t-test was used to determine that the cohorts did not vary significantly in age (*p* = 0.658). Additionally, a Fisher’s exact test revealed that the cohorts did not vary significantly in sex (*p* = 0.416), and race (*p* = 0.715).

Patients met the following eligibility criteria: (1) adults (18+) with a diagnosis of adductor (*n* = 20) or abductor laryngeal dystonia (*n* = 3), or both (*n* = 1), (2) symptomatic during research participation, (3) at least 3 months post-Botox injection, and (4) native English speakers. Additionally, patients did not demonstrate significant cognitive impairments on informal assessment or notable hearing deficits on formal auditory screening. Participants incapable of providing informed consent, with current or history of neurological disorders, or other forms of dystonia were excluded from the study. Healthy controls had no history of physiological brain abnormalities, otolaryngological problems, or neurological illness. All studies were approved by the UCSF Institutional Review Board, and participants provided informed consent prior to participation (see Table 1).

**Table 1:** Participant demographics.

Demographics	Controls	LD patients	*p* value
Sample size	29	24	–
Age	55.6 ± 14.6	54.1 ± 10.5	0.658^a^
Sex	16 Female	16 Female	0.416^b^
Race	24 White, 5 Unknown	21 White, 1 Black, 1 Other, 1 Unknown	0.715^c^
Diagnosis	–	20 ADLD, 3 ABLD, 1 ABLD/ADLD	–

a Welch’s two sample *t* test.

b Fisher’s exact test measuring female vs male.

c Fishers exact test measuring white vs non-white race.

### 2.2 Instrumentation and experimental design

The experiment was conducted inside a magnetoencephalography (MEG) scanner (CTF, Coquitlam, BC, Canada), where participants reclined in a supine position. We focus on the behavioral and psychophysics data in this manuscript and will publish the imaging data in a separate manuscript. Vocalizations were captured using a MEG-compatible optical microphone (Phone-Or Ltd., Or-Yehuda, Israel) and processed through a digital signal processing (DSP) system. Auditory feedback was delivered via insert earphones (ER-3A, Etymotic Research, Inc., Elk Grove Village, IL, USA), with a net delay of 19 ms (Kim et al., 2024).

Prior to the experiment, the auditory input volume was adjusted to a comfortable level to approximate natural speech perception without earphones. Participants completed 74 trials, each beginning with the presentation of a visual cue—a green dot displayed on a screen in front of them—prompting them to vocalize the vowel /a/ for 2.5 s. After a randomly jittered delay of 200–500 ms from phonation onset, the DSP altered the pitch by +100 or −100 cents (1/12th of an octave) for 400 ms. However, for this study, only the first 200 ms before phonation onset, prior to any pitch perturbations, were analyzed. Following each trial, the screen remained blank for 2.5 s, ensuring no stimulus or vocalization occurred. Optional breaks were provided after every 15 trials to maintain participant comfort.

### 2.3 Data preprocessing and analysis

An autocorrelation window-based pitch tracking method was applied to derive a pitch-time-course in Hertz from the raw acoustic recordings. A minimum signal-to-noise ratio threshold was identified for each trial and applied to ensure accurate detection of voice onset and to filter out pitch tracking errors. Trials were automatically excluded if the phonation did not last the entire duration of the trial or in case of low-quality pitch tracks. Additional trials were excluded manually with visual inspection due to the presence of excessive noise or due to incorrect phonation (human error). In all such cases, trials were only rejected, and no artifact removal methods were used to modify any of the signals. As a result of these procedures, 25.32% of trials were excluded for the LD group and 7.74% for the control group. The higher proportion of excluded trials in the LD group is consistent with the increased acoustic instability associated with disordered speech in LD and were excluded to ensure that unreliable acoustic signals did not influence the results. Of these remaining trials, any outliers (where the initial pitch is over two standard deviations away from the group mean initial pitch) were excluded from the analysis. All analyses were performed using acoustic data organized in MATLAB structs, before being ingested into Python dataframes to compute pitch centering metrics and perform trial-level analyses.

To account for individual differences in absolute pitch range, all pitch values were converted from hertz to cents. This conversion was performed by normalizing the pitch at each point in time *F_trial,Hz_*(*t*) using the subject-wise median pitch at each time point *F_median,Hz_*(*t*) which served as a stable within-subject baseline (Equation 1).


$$F_{\text{trial},\text{cents}}(t)=1200\;\log_{2}(\frac{F_{\text{trial},\mathrm{Hz}}(t)}{F_{\text{median},\mathrm{Hz}}(t)})$$ (1)

**Figure 1:**
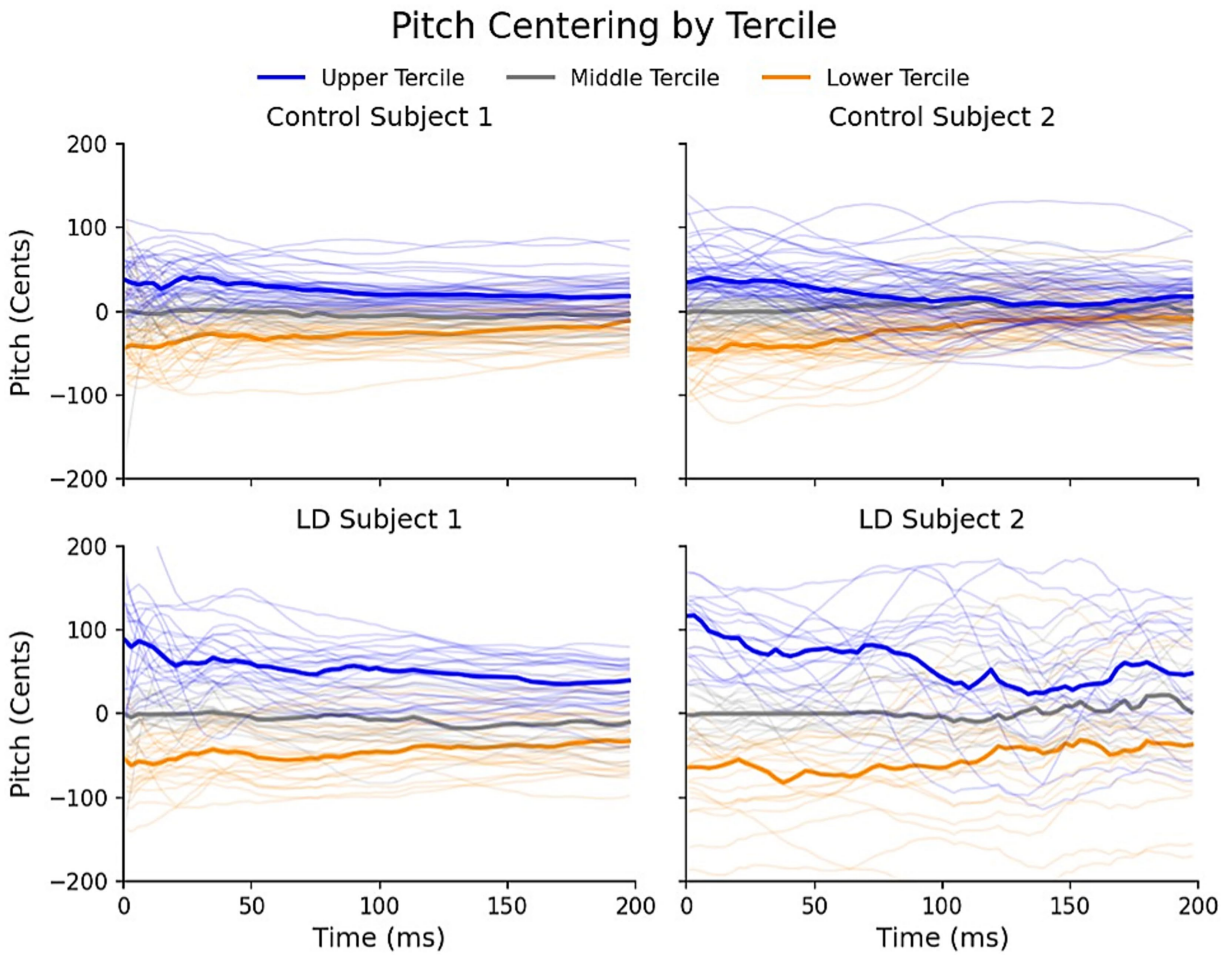
Tercile-based visualization of pitch centering in cents. Individual trial-level pitch centering measurements are shown for two controls and two participants with laryngeal dystonia (LD), grouped into lower, middle, and upper terciles based on pitch centering values. Tercile distributions are completed within the first 50 ms after voice onset. Thicker bars represent the median centering within each tercile for each participant. The figure illustrates pitch convergence toward the median as the utterance production progresses.

Trials were categorized into one of three tercile groups based on the initial pitch distribution within a subject: lower tercile (0–33.3rd percentile), central tercile (33.4–66.5th percentile) and upper tercile (66.6–100th percentile). Subjects with a trial count of less than 25 were excluded from subsequent analyses. For the remaining subjects, we computed the trial-wise “initial pitch” (the mean pitch from 0 to 50 ms) and “mid-trial pitch” (the mean pitch from 150 to 200 ms). Trial-level visualizations further illustrate the observed pitch centering behavior in individuals with LD and healthy controls (Figure 1): pitch values show greater deviation from the median during the initial 0–50 ms window, than they show in the subsequent 150–200 ms time window, suggesting a convergence toward the median as the utterance production progresses.

**Figure 2:**
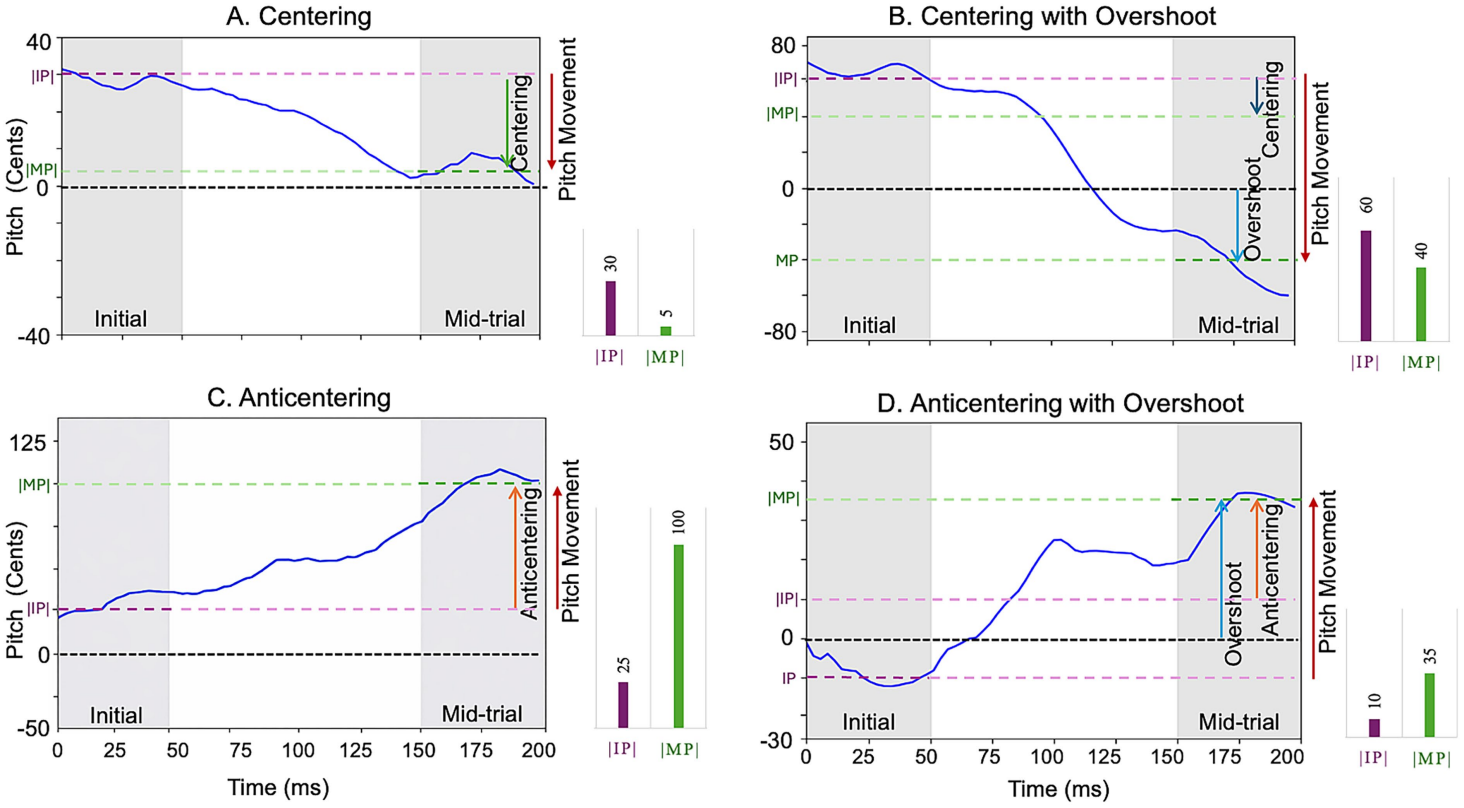
Pitch trajectories illustrating distinct centering behaviors. Example pitch traces from individual trials are shown to demonstrate the range of centering patterns observed. The pink line corresponds to the median pitch at the onset of an utterance (0–50 ms) and the green line corresponds to the median pitch between 150–200 ms after onset. Each panel represents a different trial type: **(A)** centering (pitch shift toward the median pitch), **(B)** centering with overshoot (pitch shift toward the median pitch, and where the normalized pitch movement crosses the median at mid-trial), **(C)** anticentering (pitch shift away from the median pitch), and **(D)** anticentering with overshoot (pitch shift away from the median pitch, and where the normalized pitch movement crosses the median at mid-trial).

All downstream analyses were computed using the initial pitch and mid-trial pitch of each trial (Figure 2). We first computed centering and pitch movement, the primary outcome measures of this study, for each trial. Centering was defined as the difference in absolute values of initial and mid-trial pitch. Hence positive values (centering > 0) indicated a shift toward the median pitch resulting in “centering” trials (Figures 2A,B), whereas negative values (centering < 0) reflected a movement away from it resulting in anti-centering trials (Figures 2C,D). Pitch movement was calculated as the difference between mid-trial and initial pitch. To ensure consistency across conditions, normalized pitch movement for trials in the upper tercile was computed by reversing the sign of pitch movement. This standardization allowed for meaningful comparisons between different terciles of initial pitch. Additionally, a subset of trials was classified as overshoot trials (Figures 2B,D), defined as instances where the normalized pitch movement crosses the median pitch at mid-trial. In these trials, the pitch shifted beyond the median reference point, such that the total pitch movement included both the centering component and an additional overshoot component. Hence, every trial was classified by tercile (upper/central/lower), centering type (centering/anti-centering), and overshoot (overshoot/non-overshoot).

### 2.4 Statistical analyses

We developed a Python analysis pipeline to generate a trimmed dataframe, which was then imported into R (version 4.2.2) (R Core Team, 2021) for further statistical analysis. To conduct the analyses while accounting for variability at the trial level, all trials were included and pooled across subjects within each group, with subject identity retained. Importantly, we limited our comparisons to the lower and upper terciles, as the central tercile tends to include lower-magnitude initial pitch values by design, which could lead to artificially reduced perceived errors and bias in centering magnitude comparisons. A linear mixed effects model was used to examine the main effects of group (LD vs. Controls), tercile (lower vs. upper), and their interaction on initial pitch deviation, pitch movement magnitude, and pitch centering. Trial-level observations were treated as repeated measures nested within each subject. We also assessed within-group differences between terciles (lower vs. upper).

The model can be expressed as:


$$Y_{\mathrm{it}}=\beta_{0}+\beta_{1}{\text{Group}}_{i}+\beta_{2}{\text{Tercile}}_{\mathrm{it}}+\beta_{3}({\text{Group}}_{i}\times {\text{Tercile}}_{\mathrm{it}})+\varepsilon_{\mathrm{it}}$$

where $Y_{\mathrm{it}}$ denotes the outcome for trial t of subject i , and $\varepsilon_{\mathrm{it}}$ represents trial-level residual error. Model assumptions were evaluated by inspection of residual distributions and residual-versus-fitted plots. Residuals were approximately centered around zero with stable variance.

Descriptive statistics, including frequencies, percentages, and standard errors, were computed for each trial type. Trial type frequencies were summarized as proportions and reported as percentages by group. Additional statistical analyses were performed using SAS version 9.4. (SAS Institute Inc., Cary, NC) at two-sided type 1 error rate of 0.05.

## 3 Results

### 3.1 Pitch behaviors across all trials

#### 3.1.1 Initial pitch deviation and pitch movement magnitude were greater in individuals with LD compared to healthy controls across all trial types

**Figure 3:**
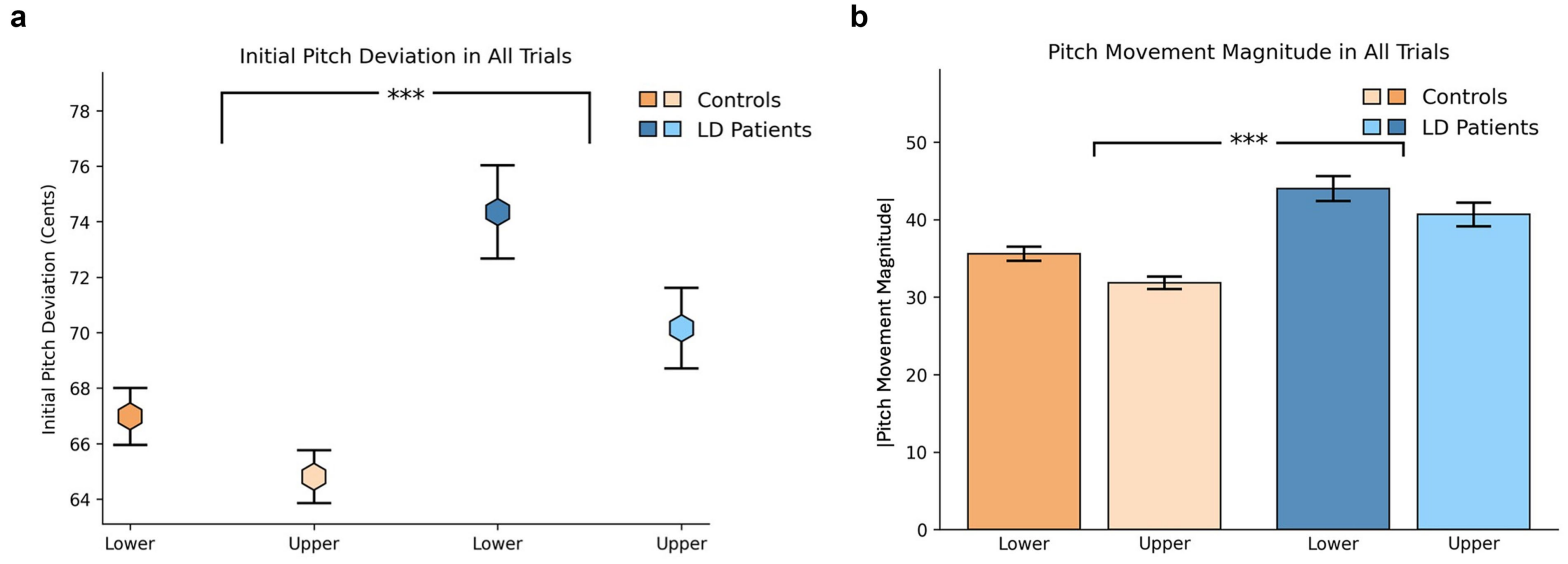
**(a)** Initial pitch deviation across all trial types. The hexagonal dots represent mean values in cents, with standard error indicating variability. Significant group and tercile differences observed. **(b)** Pitch movement magnitude (difference between mid-trial and initial pitch) across all trial types. Mean absolute pitch movement magnitude (in cents) is shown, with error bars representing standard error. Significant group and tercile differences observed.

Initial pitch deviation, defined as the deviation from the median pitch at trial onset, was significantly greater in the LD group compared to controls (LS-mean difference = 6.36, 95% CI [3.82, 8.89], *p* < 0.0001), with tercile differences noted (3.18, [0.64, 5.72], *p* = 0.015) (Figure 3a). Similarly, pitch movement magnitude, calculated as the change in pitch from onset to mid-trial, was significantly higher in LD patients than in controls across all trial types (8.63, [6.31, 10.95], *p* < 0.0001), with significant tercile-related variation present in both groups (3.55, [1.22, 5.87], *p* = 0.004) (Figure 3b).

#### 3.1.2 Individuals with LD demonstrated greater centering magnitude compared to healthy controls across all trial types

**Figure 4:**
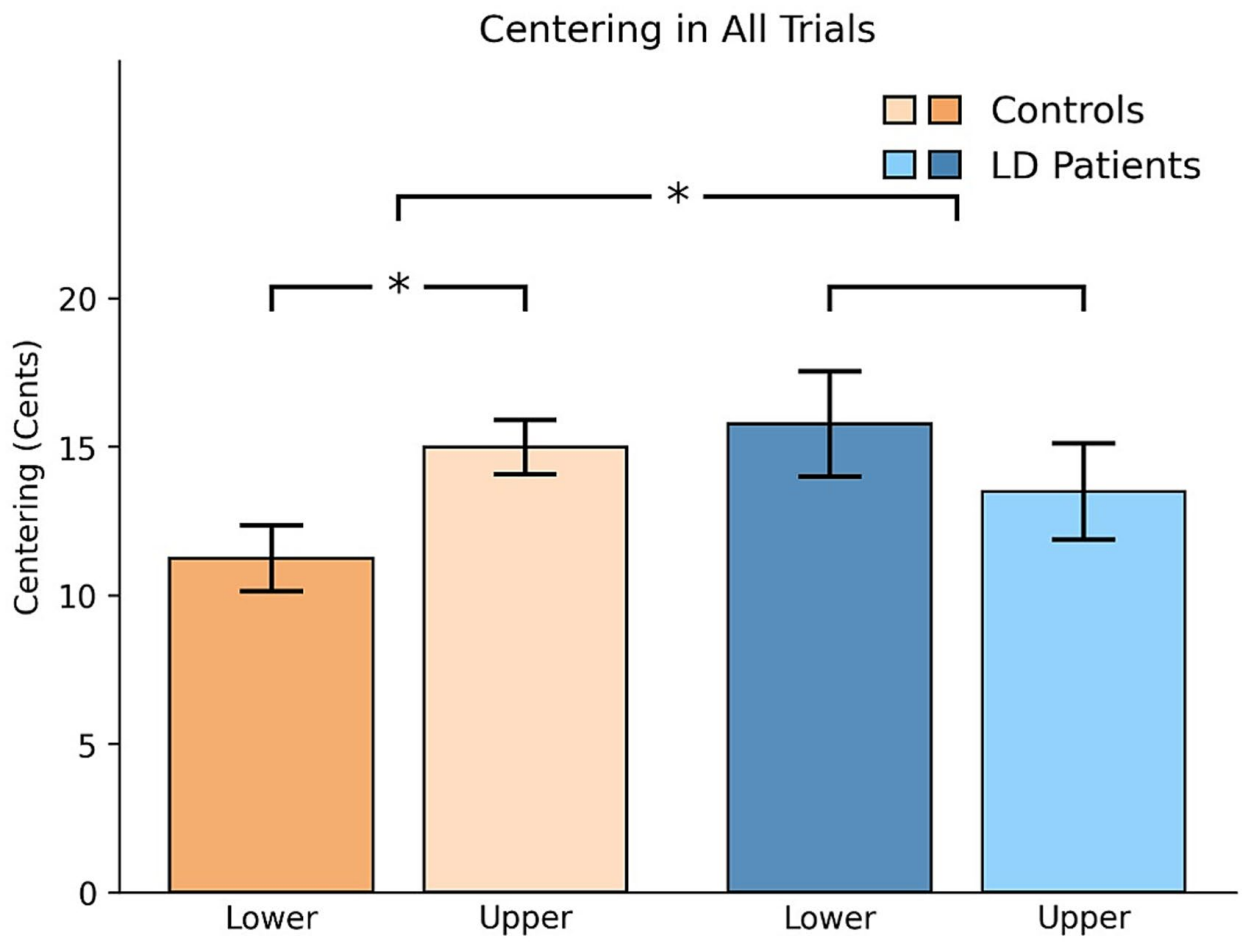
Pitch centering magnitude (difference in the absolute values of initial and mid-trial pitch) across all trial types. Mean centering magnitude (in cents) is shown, with error bars representing standard error. Significant group × tercile interaction and post-hoc within-tercile differences in the control group observed.

Patients with LD demonstrated greater centering magnitude compared to healthy controls, with a significant group-by-tercile interaction (*p* = 0.028). *Post hoc* analysis further revealed a significant difference between the upper and lower terciles within the healthy control group (3.74, [0.64, 6.84], *p* = 0.019) (see Figure 4).

Two sensitivity analyses were conducted to evaluate the robustness of the main findings. Firstly, the primary findings were replicated when analyses were restricted to participants with adductor laryngeal dystonia (ADLD), excluding those with abductor involvement (*n* = 4), with key findings remaining statistically significant. Additionally, to address differential trial exclusion between groups (25.32% vs. 7.74%), a sensitivity analysis was performed in which ~18% of control trials were randomly excluded and results for initial pitch deviation remained unchanged.

### 3.2 Pitch behaviors by trial types

#### 3.2.1 Individuals with LD and controls showed similar distributions of centering and anticentering trial types

When trials were categorized by trial type, the overall distribution of centering and anticentering trials was similar between individuals with LD and healthy controls. Controls demonstrated 67.4% centering trials, while individuals with LD demonstrated 67.26%. Likewise, anticentering trials occurred in 32.6% of control trials and 32.74% of LD trials. However, differences emerged within the overshoot subset. In *centering with overshoot* trials, LD participants exhibited a higher proportion (15.8%) compared to controls (12.9%). A similar pattern was observed in *anticentering with overshoot* trials, with LD participants showing 3.6% and controls showing 2.4%. No significant differences in the proportion of trials across trial type or across group were found.

#### 3.2.2 LD patients exhibited significantly greater centering magnitude across trial types compared to healthy controls

**Figure 5:**
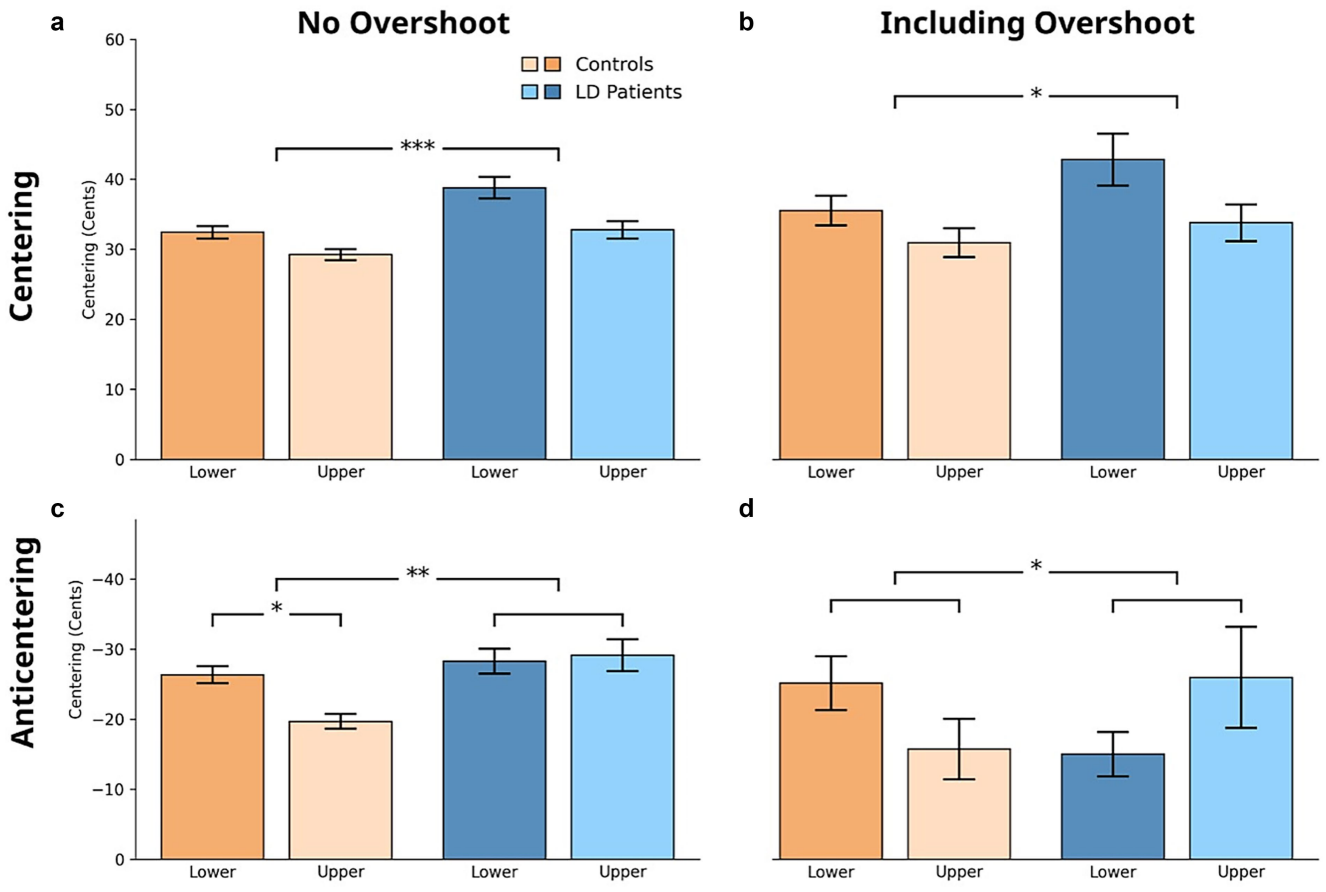
Pitch centering magnitude (difference between mid-trial and initial pitch) across: **(a)** centering trials (significant group and tercile differences observed), **(b)** centering with overshoot trials (significant tercile differences observed), **(c)** all anticentering trials (significant group differences and group × tercile interaction observed), and **(d)** anticentering with overshoot trials (significant group × tercile interaction observed). Mean centering magnitude (in cents) is shown, with error bars representing standard error.

Analyses of centering magnitude (in cents) revealed significant differences between groups. Across all centering trials, LD patients exhibited greater centering magnitude than controls (4.94, [2.74, 7.13], *p* < 0.0001), with significant effects of tercile as well (4.62, [2.43, 6.81], *p* = 0.0001). The significance of the results remained unchanged when initial pitch was included as a covariate in the statistical analysis. Additionally, within the subset of centering with overshoot trials, a significant tercile (6.82, [1.63, 12.02], *p* = 0.011) effect persisted. In anticentering trials, individuals with LD showed significantly greater anticentering magnitude than controls, with significant main effects of group (5.68, [2.55, 8.81], *p* = 0.001), and a group and tercile interaction (*p* = 0.019). *Post hoc* analysis further revealed a significant difference between the upper and lower terciles within the healthy control group (6.67, [3.00, 10.34], *p* = 0.001). Within the overshoot subset of anticentering trials, a significant group and tercile interaction was observed (*p* = 0.044), with further *post hoc* analysis revealing no significant differences (see Figure 5).

## 4 Discussion

In this study, individuals with LD exhibited significantly enhanced pitch centering during centering trials compared to healthy controls. This pattern was also observed in anticentering trials, where individuals with LD demonstrated significantly greater anticentering magnitudes compared to healthy controls.

### 4.1 Neural and behavioral findings in laryngeal dystonia

The enhanced centering found in individuals with LD may stem from neural motor control abnormalities of this neurological voice disorder. Neurophysiological evidence indicates that individuals with laryngeal dystonia exhibit reduced intracortical inhibition, characterized by shortened cortical silent periods originating from the laryngeal motor cortex (Chen et al., 2020). Complementary fMRI studies have shown hyperactivation in motor regions during phonation tasks, suggesting reduced efficiency and impaired inhibitory control in neural systems responsible for regulating voice during symptomatic speech (Chen et al., 2020). Additional neuroimaging studies show pre-phonatory activity in auditory processing regions, such as the superior and middle temporal gyri, in individuals with LD. This suggests that abnormalities in the generation of auditory predictions may emerge even before voice onset (Kothare et al., 2022; Simonyan and Ludlow, 2010). These imaging findings broadly support the presence of disinhibition and hyperactivity in the speech motor system of individuals with laryngeal dystonia, potentially contributing to pitch centering impairments.

Behaviorally, in this study cohort, LD patients present with larger initial pitch deviations compared to controls. Previous studies report similar observations when comparing the centering between central (reduced centering) and peripheral (enhanced centering) trials (Subrahmanya et al., 2024; Niziolek et al., 2013). However, despite this hypothesized compensatory behavior, individuals with LD also demonstrated greater overshoot magnitudes in this study, suggesting that these corrections may not be optimally regulated. This may potentially be due to impaired motor and somatosensory control inherent to their neurological voice disorder in this patient population. Individuals with LD exhibit altered vocal tone and increased laryngeal tension, which may influence the variability and trajectory of their pitch responses. For instance, the enhanced pitch centering observed in lower-tercile trials in our results may reflect a hyperfunctional voice pattern or a compensatory effort to maintain pitch stability. Importantly, the direction of pitch change, whether raising or lowering, does not fully account for the group differences in centering magnitude, as the main effect of group remains consistent across trial types. Lastly, sensitivity analyses excluding participants with abductor involvement or equalizing trial counts between groups did not alter the pattern or statistical significance of the main findings, indicating that the observed centering behavior is robust. This suggests that these effects primarily reflect characteristics of LD, particularly the adductor subtype, which represents the predominant clinical phenotype.

Similar impairments have been noted in neurodegenerative conditions such as Alzheimer’s disease (AD), where patients exhibited greater centering responses in the lower tercile than in the upper tercile (Subrahmanya et al., 2024), which is consistent with our findings in both the control and LD populations. Furthermore, like patients with AD, patients with LD showed greater initial pitch deviations than controls in this study. However, the behaviors were not identical: while the AD study showed comparable initial pitch deviations across upper and lower terciles, patients with LD demonstrated greater pitch deviations for the lower tercile compared to the upper tercile. Pitch centering may be impaired in neurological disorders involving sensorimotor integration deficits; however, further research is needed to characterize the specific characteristics of pitch centering across various clinical populations with voice and motor speech disorders.

### 4.2 Integration with computational modeling frameworks

Our results align with computational modeling frameworks, such as the State Feedback Control (SFC) model of speech motor control (Houde and Nagarajan, 2011). In healthy speakers, pitch centering is thought to reflect an active process aimed at stabilizing vocal output and regulating pitch toward an intended target pitch—a modeling-based hypothesis analogous to the phenomenon of a median pitch, which is purely empirical. The discrepancy between initial pitch and the target pitch, referred to as target error, is a primary driver of centering behavior. Target error may stem from inaccuracies in the feedforward pathway, resulting from motor planning noise or imprecise internal estimation of the target. These discrepancies may be detected via efference copy, which generates immediate predictions about the estimated state and can be compared to the intended target state. These comparisons would result in successive corrective updates to the controls through the feedforward control system. With regard to feedback mechanisms, while the specific contribution of auditory feedback in pitch centering remains unclear (Parrell et al., 2021), somatosensory feedback may play a role. Variability in initial pitch could be introduced by controller noise leading to large target errors. Once this deviation is detected, the system responds with corrective motor adjustments to bring pitch closer to the intended target, resulting in centering. The magnitude of centering responses is partially influenced by the size of the initial pitch deviation, such that greater target errors are likely to elicit stronger corrective or compensatory responses. In this framework, larger centering magnitudes may reflect the need to traverse a greater perceptual-motor space to reach the intended vocal target.

As predicted by the SFC model, the increased controller gain, superimposed by the greater initial pitch deviation seen in individuals with LD in this study cohort, may cause the system to generate stronger corrective movements in response to pitch deviations, contributing to the presence of enhanced pitch centering and overshoot observed in our findings. Similar results have been identified in individuals with focal task-specific dystonia, with abnormally elevated gain within the sensorimotor loop leading to heightened activity in sensory cortical regions. This may contribute to unstable motor output explaining dystonic features such as muscular co-contraction and overflow movements (Sanger and Merzenich, 2000). In addition to elevated controller gain, the greater anticentering magnitude observed in individuals with LD in our study may arise from increased variability, or controller noise, within the speech motor system. Heightened controller noise can produce inconsistent or misdirected responses, leading to large deviations away from the intended pitch target. This suggests that anticentering behavior may stem from poor motor execution. This behavior bears resemblance to “following” pitch trajectories observed in response to external pitch perturbations, wherein the speaker’s pitch shifts in the same direction as the perturbation, opposite to what would be expected if compensating for the externally introduced feedback error (Behroozmand et al., 2012). While we categorized trials into centering and anticentering types, it is possible that these may reflect a continuum of motor control responses rather than distinct mechanisms. The similarity in group differences across both trial types may support a unified control model, rather than one in which different strategies or systems are engaged for different trial types. This hypothesis aligns with findings from prior studies which suggest that following responses may not represent a distinct response pattern, but the tail of a unimodal distribution (Miller et al., 2023).

## 5 Future directions

This study is the first to investigate pitch centering in individuals with laryngeal dystonia. While the current sample focused primarily on individuals with the adductor subtype, future work should include larger cohorts and extend to additional subtypes, such as abductor laryngeal dystonia, vocal tremor, and patient populations with co-occurring vocal tremor and laryngeal dystonia. To further understand the underlying mechanisms, future studies should also incorporate neuroimaging techniques, particularly magnetoencephalography (MEG), to directly examine the neural processes involved in pitch centering. Moreover, broader disease characterization will be essential for understanding the neural correlates and subtype-specific patterns of pitch regulation in this population.

Overall, these findings underscore the complexity of feedforward and feedback-based vocal control in LD and highlight pitch centering as a sensitive behavioral index of disrupted sensorimotor integration. Future work is needed to further characterize the mechanisms driving enhanced pitch centering and to determine whether they reflect adaptive compensation, maladaptive motor speech control, or a combination of both. Greater understanding about pitch centering in individuals with LD can inform clinical assessment, diagnoses, and use of potential treatment outcomes that would complement acoustic and perceptual measurements of speech.

## Data Availability

The datasets and analysis pipelines presented in this study can be found in the following online repository: https://osf.io/5umr4/overview?view_only=0a00d3a88ccc4d6f9534ed04bfa7ee74.
